# Cytokine Signaling in Diabetic Neuropathy: A Key Player in Peripheral Nerve Damage

**DOI:** 10.3390/biomedicines13030589

**Published:** 2025-02-28

**Authors:** Zahra Nashtahosseini, Majid Eslami, Elham Paraandavaji, Alireza Haraj, Bahram Fadaee Dowlat, Ehsan Hosseinzadeh, Valentyn Oksenych, Ramtin Naderian

**Affiliations:** 1 Department of Biology, University of Guilan, Rasht 41996-13776, Iran; z_nashtahoseini@yahoo.com; 2 Cancer Research Center, Semnan University of Medical Sciences, Semnan 35147-99442, Iran; m.eslami@semums.ac.ir; 3 Clinical Research Development Center, Baharloo Hospital, Tehran University of Medical Sciences, Tehran 13399-73111, Iran; 4 Student Research Committee, Faculty of Medicine, Iran University of Medical Sciences, Tehran 14496-1453, Iran; 5 Faculty of Medicine, Iran University of Medical Sciences, Tehran 14496-1453, Iran; 6 Department of Surgery, School of Medicine, Semnan University of Medical Sciences, Semnan 35147-99442, Iran; 7 Faculty of Medicine, University of Bergen, 5020 Bergen, Norway; 8 Clinical Research Development Unit, Kowsar Educational, Research and Therapeutic Hospital, Semnan University of Medical Sciences, Semnan 35147-99442, Iran

**Keywords:** cytokine signaling, diabetic peripheral neuropathy (DPN), peripheral nerve damage, inflammation, biomarker

## Abstract

Diabetic peripheral neuropathy (DPN) is a debilitating complication of diabetes mellitus, characterized by progressive nerve damage driven by chronic hyperglycemia and systemic inflammation. The pathophysiology of DPN is significantly influenced by pro-inflammatory cytokines, such as IL-1β, IL-6, and TNF-α. These cytokines promote oxidative stress, vascular dysfunction, and neuronal degeneration by activating important signaling pathways including NF-κB and MAPK. While IL-6 promotes a pro-inflammatory microenvironment, increasing neuronal damage and neuropathic pain, TNF-α and IL-1β worsen Schwann cell failure by compromising axonal support and causing demyelination. Immune cell infiltration and TLR activation increase the inflammatory cascade in DPN, resulting in a persistent neuroinflammatory state that sustains peripheral nerve injury. The main characteristics of DPN are axonal degeneration, decreased neurotrophic support, and Schwann cell dysfunction, which weaken nerve transmission and increase susceptibility to damage. Advanced glycation end-products, TNF-α, and CXCL10 are examples of biomarkers that may be used for early diagnosis and disease progression monitoring. Additionally, crucial molecular targets have been found using proteomic and transcriptome techniques, enabling precision medicine for the treatment of DPN. This review emphasizes the importance of cytokine signaling in the pathogenesis of DPN and how cytokine-targeted treatments might reduce inflammation, restore nerve function, and improve clinical outcomes for diabetic patients.

## 1. Introduction

Diabetic peripheral neuropathy (DPN) is a severe condition caused by diabetes mellitus (DM), a disorder that affects a large number of people. This occurs when nerve injury causes painful and incapacitating symptoms. Research has highlighted how crucial cytokines, proteins involved in inflammation, are in developing DPN [1]. These markers can be linked to injury in the peripheral nerves, shedding light on how inflammation plays a role in this condition [1,2]. DM is associated with damage to target organs, particularly the microvascular consequences of retinopathy, neuropathy, and nephropathy. The United States incurs around USD 245 billion in healthcare expenses yearly due to the considerable costs of managing these illnesses [3]. DPN is a severe and prevalent complication of diabetes that is defined by nerve damage that is the consequence of persistently elevated blood sugar levels. It impacts a substantial number of individuals with diabetes, with estimates indicating that up to 50% of diabetic patients may develop some neuropathy throughout their lives [4]. The primary attribute of diabetic neuropathy is the damage or malfunctioning of nerve fibers; nonetheless, its symptoms are variable. Painful diabetic peripheral neuropathy (pDPN) is a condition that significantly impacts both health-related quality of life and general functionality, affecting around 20% of patients [5].

Cachectin, also known as tumor necrosis factor-alpha (TNF-α), is a pro-inflammatory cytokine critical in developing diabetic neuropathy. This process is distinct from other diabetes-related problems, such as retinopathy and nephropathy, in which interleukin-6 (IL-6) and C-reactive protein (CRP) play a more prominent role. The production of TNF-α by macrophages is induced by chronic hyperglycemia, which exacerbates insulin resistance, promotes atherosclerosis, and contributes to the demyelination of Schwann cells. This demyelination ultimately leads to peripheral neuropathy, which causes pain [6,7,8]. Cytokines are small proteins secreted by various cells, including immune cells, Schwann cells, and glial cells. They play a significant role in mediating inflammatory responses. In the context of DPN, pro-inflammatory cytokines such as TNF-α, interleukins (IL-1, IL-6), and chemokines are particularly important. These cytokines are produced in response to hyperglycemia and other metabolic disturbances associated with diabetes [1].

Pro-inflammatory cytokines, including TNF-α, IL-1β, and IL-6, are critical drivers in the development of diabetic neuropathy, but chemokines and anti-inflammatory cytokines also play a substantial role in modulating the disease. Chemokines, a specialized group of cytokines responsible for immune cell recruitment, can have both harmful and protective impacts on diabetic neuropathy progression. For example, monocyte chemoattractant protein-1 (MCP-1/CCL2) has been linked to neuroinflammation by promoting macrophage infiltration into peripheral nerves, intensifying oxidative stress, and impairing Schwann cell functionality. Likewise, CXCL10, a chemokine activated by TNF-α and IFN-γ, aggravates neuroinflammation by attracting T cells, which escalate immune-related damage to peripheral nerves. Elevated levels of CXCL10 have been associated with greater DN severity, underlining its potential as both a biomarker and a target for therapeutic intervention [9]. Anti-inflammatory cytokines play a vital role in regulating neuroinflammation by limiting immune activation and promoting neuronal survival. IL-10, a powerful immunoregulatory cytokine, inhibits NF-κB activation and suppresses the production of TNF-α and IL-6, thereby mitigating inflammation-induced neuronal damage. Similarly, IL-4 promotes macrophage polarization toward the M2 phenotype, which helps alleviate oxidative stress and supports nerve repair. TGF-β, another prominent anti-inflammatory cytokine, influences Schwann cell activity, reduces excessive infiltration of immune cells, and drives extracellular matrix remodeling essential for nerve regeneration. Nevertheless, its dual function—enhancing axonal repair while potentially inhibiting remyelination—demands a carefully balanced therapeutic strategy [10].

Emerging therapeutic approaches focusing on chemokines and anti-inflammatory cytokines hold significant potential in managing diabetic neuropathy. Inhibiting CCL2/CCR2 signaling through pharmacological agents has shown neuroprotective benefits by reducing macrophage-driven inflammation. Furthermore, IL-10-based treatments have been studied for their role in restoring immune homeostasis. Recombinant IL-4 and TGF-β modulators have also been explored for their potential to improve Schwann cell functionality and preserve axonal integrity. Gaining a thorough understanding of the dynamic interaction between pro-inflammatory and anti-inflammatory mediators is crucial for designing precision-targeted strategies to curb the progression of neuropathy in diabetes [11].

### Mechanisms of Action Include

(1) Inflammatory Cascade Activation: The activation of signaling pathways such as nuclear factor-kappa B (NF-κB) leads to increased expression of pro-inflammatory cytokines. This cascade contributes to oxidative stress and neuronal damage [1]. (2) Neuronal Sensitization: Cytokines can sensitize neurons at the first pain synapse, enhancing pain perception and contributing to neuropathic pain [12]. (3) Neuroimmune Interactions: Cytokines facilitate communication between neurons and non-neuronal cells, which is crucial for understanding neuropathic pain mechanisms [12].

A chronic condition characterized by low-grade inflammation occurs in diabetes, marked by elevated levels of pro-inflammatory cytokines such as TNF-α, IL-1, and IL-6. Cytokines are integral to the body’s response to oxidative stress; however, excessive levels of these signaling molecules can compromise neuronal health and function. This dysregulation may result in detrimental effects on overall brain health [1]. The infiltration of immune cells, including macrophages and T-cells, within peripheral nerves has been documented in individuals with diabetes. This immune response results in increased inflammatory markers, exacerbating neuropathy’s progression and highlighting the necessity for targeted interventions [1]. The activation of pattern recognition receptors (PRRs), such as Toll-like receptors (TLRs), further amplifies inflammatory signaling pathways, worsening nerve damage [1]. Elevated levels of inflammatory factors correlate with the severity of DPN. For instance, studies have shown that specific chemokines like MCP-1 and CCL3 are positively associated with tactile perception thresholds in diabetic patients, indicating a direct relationship between inflammation and sensory nerve damage [13]. Inflammatory cytokines induce ischemia of peripheral neurons by increasing oxidative stress and damaging blood vessels. This process contributes to the development of neuropathic pain and exacerbates neuronal damage [14,15]. Neurodegeneration can be induced by the prolonged inflammatory response through mechanisms such as mitochondrial failure and endoplasmic reticulum stress. Ultimately, these pathological alterations exacerbate the sensitivity to damage and impede neuronal function [1,14,16].

## 2. Cytokines and Their Role in Inflammation and General Mechanisms of Cytokine Signaling in Inflammatory Conditions

Inflammation is an important process triggered by several mediators, like physical, chemical, and biological factors and mechanical tissue damage. Its characteristic features include the dilation of veins, increased vascular permeability, and the infiltration of inflammatory components, all of which play an essential role in the body’s response to injury or infection [17]. Based on duration, inflammation is classified into three types: acute, chronic, and subacute, with the latter representing a transitional phase between acute and chronic forms. The acute phase begins immediately after injury, with cytokines, acute-phase proteins, and chemokines playing critical signaling roles. These molecules recruit neutrophils and macrophages to the injury site [18]. Cytokines, a superfamily of signaling proteins, are essential for mediating cellular communication during inflammation [19]. Prominent cytokines involved in the acute phase include IL-1β, IL-6, TNF-α, interferon-γ (IFN-γ), and IL-8 [19,20]. Prolonged inflammation is associated with increased concentrations of IL-17 and IL-18 [21]. Anti-inflammatory cytokines such as IL-4, IL-10, IL-11, IL-13, and transforming growth factor β (TGF-β) play key roles in dampening the inflammatory response [22]. Interestingly, certain cytokines like IFN-α, IL-6 (which regulates pro-inflammatory cytokine levels) and TGF-β (in synergy with IL-6) exhibit dual roles, contributing to both inflammatory and anti-inflammatory pathways. IL-1β, primarily produced by mononuclear cells and epithelial cells during inflammation, injury, and infection, induces fever, stimulates cell adhesion molecule production, activates a wide array of acute-phase response (APR) genes, and enhances lymphocyte activity. Moreover, IL-1β promotes the expression and secretion of inducible nitric oxide synthase (iNOS) and cyclooxygenase-2 (COX-2), which are instrumental in generating inflammatory mediators [23]. Similarly, IL-6 is produced by various cell types, including mononuclear phagocytes, T cells, B cells, and fibroblasts [18]. It facilitates B-cell differentiation and maturation into antibody-producing cells and induces polyclonal B-cell activation. Elevated levels of IL-6 are strongly associated with chronic inflammation [18].

TNF-α is produced by macrophages and monocytes during the acute phase of inflammation. It promotes necrosis and apoptosis through activation of the TNFR-1 signaling pathway. Triggering of TNFR-1 leads to the release of silencer of death domains (SODD) from its intracellular domain. Higher concentrations of SODD are detected by the TNF receptor-associated death domain (TRADD), which subsequently recruits receptor-interacting protein (RIP), TNF-R-associated factor 2 (TRAF-2), and Fas-associated death domain (FADD) [24]. These proteins collectively induce apoptotic activity in the cell [24]. Furthermore, TRAF-2 is predicted to activate the mitogen-activated protein kinase kinase kinase (MAPKKK) pathway, further contributing to inflammation. IL-18 is produced by monocytes, macrophages, keratinocytes, and mesenchymal cells [25]. It is essential for inducing IFN-γ, promoting cell adhesion molecules, synthesizing nitric oxide, and producing chemokines [26]. The nuclear factor-kappa B (NF-κB) pathway is one of the important pathways that are activated by the IL-18 receptor complex [27], the mitogen-activated protein kinase (MAPK) pathway in neutrophils, which stimulates iNOS expression, and the Janus kinase/signal transduction and activator of transcription (JAK/STAT, specifically STAT3) pathway, leading to IFN-γ production in neutrophils. TGF-β activity is mediated by activating its TGF-β receptor II (TGF-βRII) receptor upon ligand binding. The connection triggers kinase activity in the TGF-β receptor I (TGF-βRI) [28]. Once it is activated, TGF-βRI helps Smad proteins move, which starts the transcription of genes. This process suppresses matrix metalloproteinases and produces extracellular matrix (ECM) components. The stimulation of the p38 MAPK pathway has also been connected to TGF-β. A vital chemoattractant that guides neutrophils to inflammatory areas is IL-8. It enhances granule enzyme release and increases ROS generation [29].

Furthermore, IL-8 has been shown to exhibit potential angiogenic properties [30]. The NF-κB pathway and MAPK pathway are activated when IL-8 binds to its receptors, CXCR1 and CXCR2 [31]. This interaction also accelerates fibroblast migration and promotes fibronectin deposition. IL-17, a hallmark cytokine of T helper 17 cells, specifically its type A form, activates the MAPK pathway, stimulating IL-8 secretion [32,33] and upregulating COX-2 expression [32]. Through the JAK/STAT pathway, IFN-γ induces nitric oxide (NO) production and upregulates MHC class II expression in macrophages. It also enhances macrophage phagocytic activity by increasing reactive oxygen species (ROS) production [34]. The NF-κB pathway, JAK/STAT pathway, and MAPK pathway are the three key signaling pathways involved in inflammatory conditions [35]. The NF-κB transcription factor plays multiple roles, including regulating inflammation, immune responses, and apoptosis [36]. Under physiological conditions, IκB proteins inhibit NF-κB activation [37].

IκB kinase (IKK) activation by IL-1 and TNF-α initiates the conventional NF-κB pathway. As a result, IκB is phosphorylated and then degraded by proteases, which permits NF-κB to move into the nucleus and control the production of inflammatory genes [38]. JAK (Janus kinase) is a tyrosine kinase enzyme that mediates signaling for type I cytokines (e.g., IL-2, IL-12, IFN-γ, TNF-β) and type II cytokines (e.g., IL-4, IL-5, IL-6, IL-10, IL-13) via downstream activation of STAT transcription factors [39]. Once activated, STAT proteins bind to promoter regions of target genes, regulating the transcription of inflammatory genes [35]. IL-4, produced by activated T cells, exerts its effects by binding to IL-4 receptors (IL-4R-I and IL-4R-II) [40]. Upon receptor activation, JAK1 and JAK3 phosphorylate STAT6, while IL-4R-I also activates the PI3K/Akt pathway in association with insulin receptor substrate 2, which supports cell proliferation and survival [40]. IL-10 and IL-13 exert anti-inflammatory effects via the JAK/STAT pathway: IL-13 activates JAK1, JAK2, and TYK2, leading to STAT6 (and STAT3) activation, whereas IL-10 activates JAK2 and TYK2, stimulating STAT3 and STAT5A/B [40]. The MAPK family comprises serine/threonine protein kinases organized into three components: MAPK, MAPKK, and MAPKKK [35]. Inflammatory cytokines such as IL-1, IL-6, and TNF-α trigger MAPKKK activation. This initiates a phosphorylation cascade: MAPKKK phosphorylates MAPKK, which in turn phosphorylates MAPK. During inflammation, MAPKs activate c-Jun NH2-terminal kinases (JNK) and p38 MAPK, leading to the increased expression of inflammatory cytokines, including TNF-α [41].

## 3. Cytokine Dysregulation in Diabetes, Hyperglycemia and Immune Dysregulation

The body’s production and balance of cytokines are influenced by chronic hyperglycemia. Patients with type 2 diabetes mellitus have higher plasma concentrations of pro-inflammatory cytokines, including IL-1β, IL-6, IL-8, IL-17, IL-18, TNF-α, IFN-γ, and TGF-β, according to research (T2DM). However, the levels of anti-inflammatory cytokines, such as IL-10 and IL-13, are seen to be reduced. On the other hand, the levels of IL-4 are more contentious, with specific research showing that an increase is associated with type 2 immune responses [42,43], and others report decreased type 1 immune responses [39,42,44,45]. NLRP3 (NOD-like receptor pyrin domain 3) is an intracellular sensor that forms NLRP3 inflammasomes upon activation, leading to the maturation of IL-1β and IL-18 through caspase-1 activation [46]. TNF-α plays a significant role in this process by inducing the activation and expression of pro-IL-1β, pro-IL-18, and NLRP3, as well as promoting NLRP3 deubiquitination [46]. High blood sugar levels have been associated with increased activation of the NLRP3 inflammasome, likely due to elevated plasma glucose concentrations. TNF-α production is induced by hyperglycemia through the activation of the NF-κB [47] and MAPK pathway activation [48].

**Figure 1:**
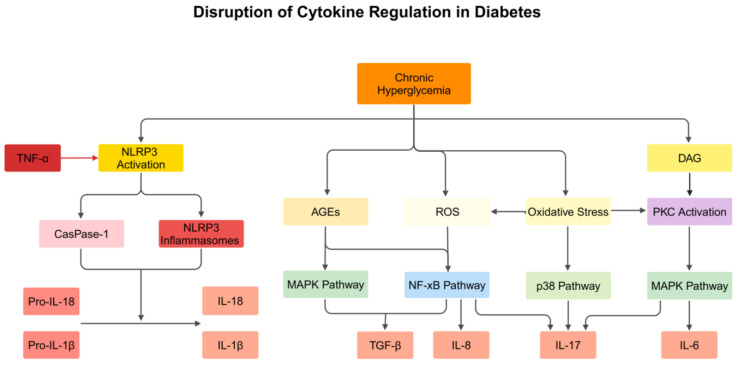
Disruption of cytokine regulation under chronic hyperglycemia conditions. Chronic hyperglycemia triggers NLRP3 activation, ROS generation, and AGEs accumulation, leading to activation of inflammatory pathways, including MAPK, NF-κB, and p38. These pathways promote the release of pro-inflammatory cytokines such as IL-6, IL-8, IL-17, IL-1β, and IL-18, contributing to systemic inflammation and diabetic complications such as neuropathy. The figure also shows the disruption of cytokine regulation in diabetes. Chronic hyperglycemia drives oxidative stress and NLRP3 inflammasome activation, leading to IL-6, IL-8, IL-17, and TGF-β upregulation through NF-κB, MAPK, and PKC pathways, amplifying inflammatory responses.

Diacylglycerol (DAG) levels are also elevated by chronic hyperglycemia due to de novo synthesis [49]. The conventional isoforms of protein kinase C (PKC-α, -β1, -β2, and -γ) are activated by DAG. Under hyperglycemic conditions, mitochondrial superoxide generation can also activate PKC. The MAPK pathway is activated by Pan-PKC activation, which stimulates the production of IL-6 [50]. Hyperglycemia results in the formation of advanced glycation end products (AGEs) and oxidative stress, which in turn promote ROS formation. These processes are linked to an increase in TGF-β, likely mediated by the activation of redox-sensitive transcription factors. For instance, AGEs binding to their receptors can activate MAPK, and both ROS and AGEs have been shown to activate NF-κB, although the latter pathway remains controversial [45,51,52]. IL-8 production is also stimulated by oxidative stress in hyperglycemic conditions through the NF-κB pathway. Similarly, IL-17 formation is stimulated through PKC, p38 MAPK, and NF-κB signaling pathways [53], as shown in Figure 1. Pro-inflammatory cytokines in the pathogenesis of diabetic peripheral neuropathy shown in Table 1.

## 4. Cytokine Signaling in Peripheral Nerve Damage

### 4.1. Inflammatory Pathways Driving Nerve Degeneration: The Role of Cytokine Signaling

TNF-α is a pro-inflammatory cytokine implicated in the development and progression of diabetic neuropathy. Peripheral nerve degeneration is facilitated by elevated TNF-α levels, which are linked to increased oxidative stress and neuronal damage. TNF-α levels and diabetic peripheral neuropathy are significantly correlated by a meta-analysis, suggesting that this protein may be a promising therapeutic target for the treatment of DN [54]. TNF-α enhances the excitability of primary afferent neurons by regulating voltage-gated sodium channels. This sensitization increases pain perception and neuropathic pain in response to nerve injury and inflammation [55,56]. Moreover, in patients with DN, elevated levels of TNF-α have been observed, correlating with reduced nerve conduction velocities and increased pain symptoms. Research shows that blocking TNF-α can improve nerve function and alleviate pain [14,57].

Elevated levels of TNF-α have been observed in patients with diabetic neuropathy, correlating with reduced nerve conduction velocities and increased pain symptoms. Studies indicate that blocking TNF-α can improve nerve function and reduce pain [14]. IL-1β is another critical cytokine that contributes to the inflammatory response in diabetic neuropathy. It is released following nerve injury and acts synergistically with TNF-α to amplify the inflammatory response [57]. Similar to TNF-α, IL-1β can induce hyperexcitability in neurons by enhancing the phosphorylation of NMDA receptors, which are crucial for pain transmission. This mechanism contributes to the development and maintenance of neuropathic pain [56]. IL-1β also promotes microglial activation, leading to further release of inflammatory mediators that perpetuate pain signaling pathways [14].

TGF-β is a multifunctional cytokine that regulates tissue repair and cell growth. It has been shown to influence Schwann cell behavior, recruit immune cells, and modulate the blood–nerve barrier’s permeability, which collectively can enhance axon regeneration after peripheral nerve injury [58,59]. However, TGF-β can also inhibit remyelination of regenerated axons, complicating its role in nerve repair [58]. It plays a dual role in peripheral nerve regeneration by promoting Schwann cell growth and recruitment while also inhibiting remyelination of regenerated axons. This complex role makes TGF-β a potential therapeutic target in treating peripheral nerve injuries [58]. After systemic damage, the CCL2/CCR2 signaling pathway regulates inflammatory responses. Muscle atrophy results from neuroinflammation and consequent injury to motor neurons, which are linked to elevated CCL2 levels. Nerve Growth Factor (NGF) signaling via its low-affinity receptor, p75, is linked to Schwann cell apoptosis, especially after axonal damage. This indicates that NGF may promote cell survival in some cases while causing cell death in others. Colony Stimulating Factor-1 (CSF-1) plays a significant role in regulating macrophage activity within peripheral nerves. The expression of CSF-1 by endo-neurial fibroblasts affects inflammation mediated by macrophages, which can worsen demyelination and nerve damage [60].

### 4.2. Mechanisms Linking Cytokines to Nerve Damage

Elevated levels of pro-inflammatory cytokines, which include TNF-α, IL-1β, and IL-6, are often observed in DPN. Hyperglycemia and other diabetes-related metabolic disorders trigger the production of these cytokines. When they are activated, a series of inflammatory responses are set off, which leads to neuronal damage and dysfunction [1,55,61,62]. TNF-α and IL-1β trigger an inflammatory response that is increased by IL-6. It contributes to prolonged neuronal damage and discomfort by maintaining the inflammatory environment that defines diabetic neuropathy [56,57]. In diabetic animals, elevated IL-6 levels have been associated with heightened pain sensitivity. The symptoms of neuropathic pain may be reduced by therapeutic measures that target IL-6 [14].

The NF-κB pathway is crucial in mediating the inflammatory response in diabetic nerves. Activation of NF-κB leads to increased expression of TNF-α and other inflammatory mediators, resulting in cellular injury, oxidative stress, and further nerve damage [1,61]. For instance, studies have shown that TNF-α can induce COX-2 expression, which is implicated in the pro-inflammatory response seen in diabetic neuropathy [61]. IL-6 mediates the expression of TNF-α and IL-1β via signaling pathways such as JAK2/STAT3 and ERK. It plays a key role in inflammation and amplifies the effects of other cytokines linked to neuropathic pain [55]. In the central nervous system, activated microglia release pro-inflammatory cytokines like TNF-α and IL-1β upon exposure to hyperglycemia or oxidative stress. This release further exacerbates neuronal damage by promoting a neuroinflammatory environment [14]. The presence of these cytokines is linked to increased neuronal excitability and pain perception, contributing to the symptoms of diabetic neuropathy [55].

Serum levels of TNF-α, IL-1β, and IL-6 are considerably greater in diabetic neuropathy patients than in healthy people, according to clinical research. Reduced sensory nerve conduction velocity is one of the markers of nerve fiber injury and dysfunction linked to these increased levels [61]. Additionally, treatment approaches focusing on these cytokines promise to improve nerve function and lower neuropathic pain [55,61]. Low doses IL-6 have been shown to enhance nerve function dramatically. This includes improved muscular action potentials and increases in the conduction velocities of sensory and motor nerves. These findings have special significance as IL-6 therapy has been associated with better nerve structure and function in mice models of DPN [63]. IL-6 may directly affect Schwann cells, which are essential for myelination and nerve repair. Promoting the growth and survival of these cells can aid in the remyelination of damaged axons and improve the overall health of the nerves [63]. Although IL-6 is typically considered a pro-inflammatory cytokine, it can also have anti-inflammatory properties. Its dual function allows it to aid in restoring homeostasis in inflammatory processes associated with diabetes [64]. IL-6 plays a role in enhancing insulin sensitivity in muscle tissues, which can be beneficial for diabetic patients. Improved glucose metabolism may indirectly support nerve health by reducing hyperglycemia-related damage [64]. Elevated levels of IL-6 have been associated with painful diabetic neuropathy compared to painless forms. This suggests that IL-6 could be a non-invasive biomarker for identifying patients at risk for painful neuropathic symptoms, aiding in early intervention strategies [65] (Table 2).

### 4.3. Cytokine-Induced Schwann Cell Dysfunction, Axonal Damage, and Neuronal Apoptosis

#### 4.3.1. Schwann Cell Dysfunction

Schwann cell dysfunction worsens with high glucose levels, impairing their support for neurons and axons due to cytoplasmic edema, reduced neurotrophic factor production, and mitochondrial impairment [66,67]. Schwann cell injury is worsened by pro-inflammatory cytokines such as TNF-α and IL-1β, which lead to inflammation and oxidative stress. When defective SCs are unable to adequately support neurons, further neuronal injury and death ensue, creating a vicious cycle of harm [67,68]. Neurotrophic factors essential for neuronal survival and regeneration are secreted by Schwann cells. In diabetes, dysfunction of Schwann cells significantly reduces the secretion of these factors, leading to axonal degradation and neuronal death [67,69].

#### 4.3.2. Axonal Damage

Schwann cells’ metabolic abnormalities cause neurotoxic intermediates to build up, which can cause direct axonal damage. The failure of SCs to provide appropriate neuronal support is intimately associated with this axonal degradation, a characteristic of diabetic neuropathy [68,69]. Impaired myelination, typified by structural anomalies in the myelin sheath, is a feature of dysfunctional Schwann cells. In addition to impairing nerve transmission, this demyelination makes the nerves more prone to damage, exacerbating neuropathic symptoms [67,70].

#### 4.3.3. Neuronal Apoptosis

**Figure 2:**
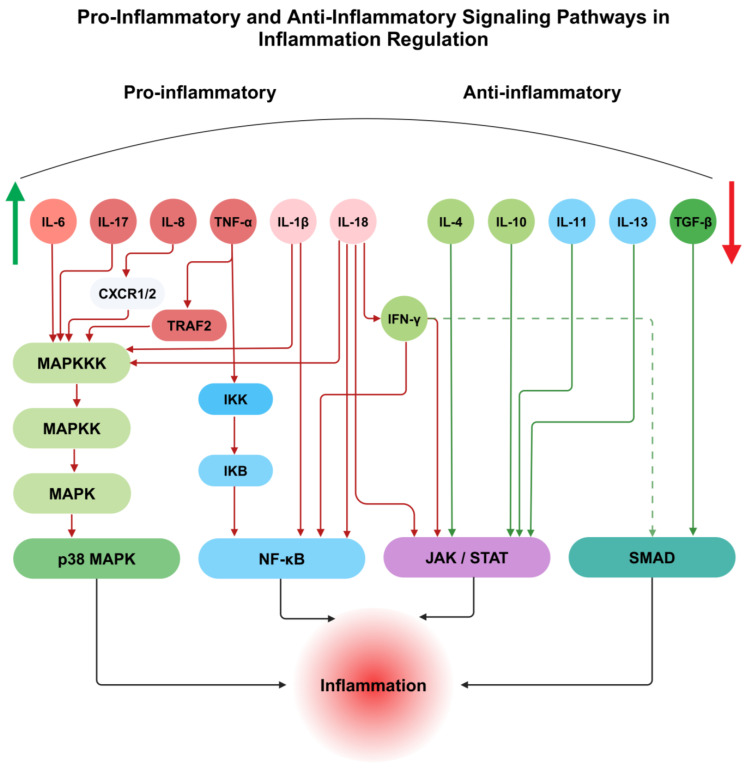
Cytokine signaling pathways in inflammation regulation. Pro-inflammatory cytokines (IL-6, TNF-α, IL-17, etc.) activate NF-κB, MAPK, and JAK/STAT pathways to drive inflammation, while anti-inflammatory cytokines (IL-10, IL-4, TGF-β) suppress inflammation through JAK/STAT and SMAD pathways. Adapted from cytokine mechanisms discussed in inflammatory neuropathies.

Elevated levels of inflammatory cytokines can trigger apoptotic pathways in neurons, initiating a cascade of cellular events that lead to cell death. This process is frequently exacerbated by oxidative stress, which results from an imbalance between free radicals and antioxidants in the body [66,70]. The loss of neuronal integrity leads to a feedback loop where damaged neurons fail to signal adequately to Schwann cells, resulting in further SC dysfunction. This cycle perpetuates both neuronal death and Schwann cell injury, worsening the overall condition of diabetic neuropathy [69], as shown in Figure 2.

## 5. Therapeutic Implications of Targeting Cytokine Signaling

### 5.1. Cytokine Modulation as a Therapeutic Strategy for Diabetic Neuropathy

Cytokines play a significant role in the pathophysiology of diabetic neuropathy, a common and debilitating complication of diabetes mellitus. Patients with diabetes that showed peripheral neuropathy had elevated levels of leptin, sE-selectin, soluble intercellular adhesion molecule (sICAM-1), soluble vascular cell adhesion molecule (sVCAM-1), CRP, fibrinogen, platelet-derived growth factor (PDGF) AB/AA, and regulated upon activation normal T cell expressed and secreted (RANTES) [71]. Mitogen-activated protein kinase-activated protein kinase 2 (MAPKAPK2), endothelial cell CD40, hypoxia-inducible factor (HIF)-1α, and phosphatase and tensin homolog (PTEN) were all found to be overexpressed. Higher osteoprotegerin was also found to be a regulator in the onset of diabetic vascular dysfunction [71]. Individuals with DPN exhibit higher concentrations of IL-6 and CRP compared to those without the condition. Administration of granulocyte-colony stimulating factor (G-CSF) to rats showed a therapeutic impact on diabetic neuropathy in a rodent model. The association of TNF-α and DPN has been proven in multiple studies [71,72]. Deletion of TNF-α specifically in proinsulin-producing bone-marrow-derived cells (PI-BMDCs) was found to be protective against DPN in mice models [72]. In contrast, epidermal growth factor (EGF) and myeloperoxidase levels were found to be lower in DPN patients [71]. Administration of EGF caused a healing effect on neuropathy and neuropathic pain in individuals [73].

### 5.2. Current Anti-Inflammatory Therapies and Their Mechanisms

Given that neuroinflammation plays a pivotal role in the pathophysiology of diabetic neuropathy, existing anti-inflammatory therapies for this condition primarily focus on mitigating its effects. Resveratrol treatment significantly reduced inflammation markers in animal models of diabetic neuropathy, including pro-inflammatory cytokines and oxidative stress indicators, and decreased pain sensitivity and improved nerve function [74]. Neuropathic pain has been demonstrated to be alleviated by non-steroidal anti-inflammatory medications (400 mg of ibuprofen four times a day). Inflammatory markers and serum levels of oxidative stress, in addition to inflammation and edema surrounding the sciatic nerve, are considerably reduced by progesterone, and are considered a protective factor in diabetic neuropathy reduction [75]. Rilmenidine helped prevent alterations in electrophysiological function and dramatically reduced sciatic nerve fibrosis and inflammation in diabetic rats. In rats given saline, sciatic nerve immunohistochemical examination revealed a decrease in nerve growth factor (NGF) and LC-3 and higher levels of HMGB-1, and TNF-α. Magnolol showed a great result in improving diabetic neuropathy in mice by reducing mitochondrial dysfunction in DRG neurons via the PPARγ/MKP-7/JNK/SIRT1/LKB1/AMPK/PGC-1α pathway and reducing inflammation through signaling of PPARγ/NF-κB [76]. Melatonin reduces oxidative stress and neuroinflammation via upregulating Nrf2 expression and NF-jB activation cascade, and decreases the high levels of COX-2, iNOS and pro-inflammatory cytokines such as IL-6 and TNF-a [77,78].

### 5.3. Novel Approaches Targeting Cytokine Pathways

**Figure 3:**
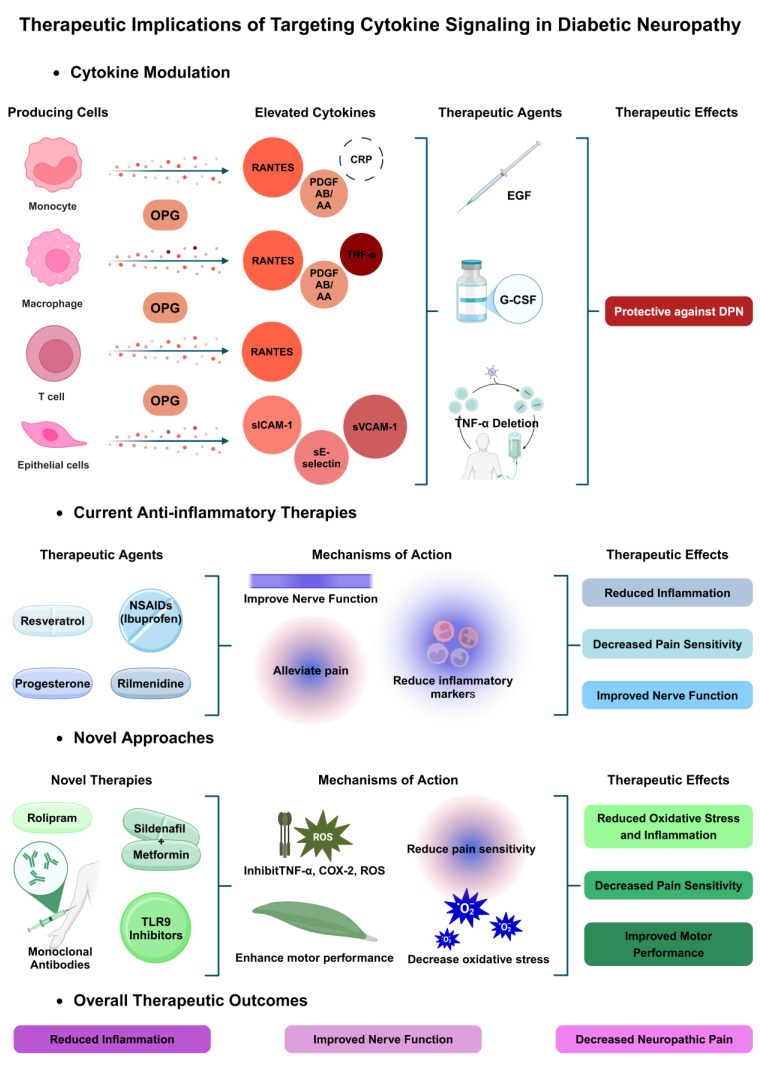
Therapeutic implications of targeting cytokine signaling in diabetic neuropathy. Current therapies and novel approaches aim to modulate cytokines (TNF-α, RANTES, sVCAM-1) and reduce oxidative stress to alleviate neuropathic pain, improve nerve function, and reduce inflammation.

For the treatment of diabetic neuropathy, new methods that target cytokine pathways have shown promise. Rolipram showed a successful decrease in oxidative stress and inflammation. The treatments inhibited the production of TNF-α and COX2, ROS, and lipid peroxidation. Additionally, enhanced motor performance was a result of NF-kB mRNA expression regulation. Severe diabetic neuropathy was reduced by TLR9 inhibition, confirming the therapeutic benefit of TLR9 targeting in the management of certain pain conditions [79]. The pairing of sildenafil and metformin may effectively reduce pain sensitivity in diabetic neuropathy patients by decreasing the activity of iNOS, which is elevated due to elevated levels of inflammatory cytokines like TNF-α and IL-6. This finding suggests that this medication combination may be a viable substitute for treating diabetic neuropathy patients’ pain. Fulranumab, tanezumab and fasinumab, which are monoclonal antibodies against the nerve growth factor, have also demonstrated efficacy in reducing pain and managing diabetic neuropathy. Ammoxetine displayed a therapeutic result by the activation of NF-κB and MAPK, which are regulators of pro-inflammatory cytokines, and reduced the production of IL-6, TNF-α, and IL-1β in diabetic rats [80], as shown in Figure 3.

## 6. Emerging Biomarkers of Cytokine-Mediated Inflammation

### 6.1. Biomarkers for Early Detection and Disease Monitoring in Diabetic Neuropathy

Early detection can help prevent progression and enable timely intervention. In a Chinese population with type 2 diabetes, patients with DPN had higher blood adiponectin levels than those without DPN [81]. Elevated variability in HbA1c levels is closely associated with DPN and may be a useful indicator of DPN in these patients. The potential function of CXCL10 was indicated when circulating levels of this chemokine were increased in diabetic patients with DPN [82]. Evaluation of AGEs with autofluorescence (sAF) has been demonstrated to be associated with diabetic neuropathy independently and have a potential use as early indicator of this condition. Nitrotyrosine (NT) and TNF-α were also regarded as possible indicators of DPN, as myelin thickness and motor and sensory nerve conduction velocities were inversely connected with sciatic nerve NT and TNF-levels [83].

### 6.2. Potential Cytokine-Related Biomarkers for Diagnosis and Treatment Efficacy

**Figure 4:**
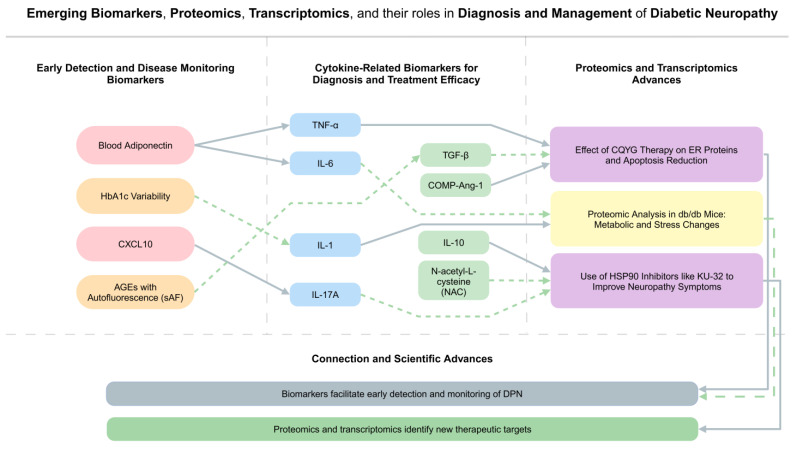
Emerging biomarkers and therapeutic advances in diabetic neuropathy. Early detection biomarkers (adiponectin, CXCL10, AGEs) and cytokine-related markers (IL-6, TNF-α, IL-10) facilitate disease monitoring. Proteomics and transcriptomics uncover novel therapeutic targets such as CQYG therapy and HSP90 inhibitors, paving the way for precision medicine approaches.

Several biomarkers associated with cytokines have been studied for their potential in diagnosis and therapy effectiveness monitoring. Elevated levels of TNF-α and IL-6 are linked to the development of some conditions, such as cardiovascular disease, obesity, and DPN, and these markers may effectively show the progression of these diseases [13]. COMP-Ang-1, by restoring molecular markers of neuropathy, increasing angiogenesis, and decreasing Cx43 and TNF-ɑ expression in the sciatic nerves of ob/ob mice, showed a new therapeutic approach related to diabetic neuropathy. In multiple studies, higher levels of cytokines such as IL6, IL10, transforming growth factor-β, IL-1, and IL-17A are also linked to increased nerve degeneration in DPN patients [71]. N-acetyl-L-cysteine (NAC), by inhibiting apoptotic markers including cytochrome c and caspase 3, may be helpful in diabetic patients with neuropathy. Virus-mediated IL-10 reduced the levels of NeuN and HSP70 protein while preventing the increase in IL-1, phosphorylated p38, TLR4, phosphorylated protein kinase C and macrophage activity. Treatment of DPN rats with salidroside reduced thermal and mechanical sensitivity by decreasing pro-inflammatory cytokines of IL-1β and TNF-α, and reducing P2X7 receptor protein production, which contributed to the mediation of neuropathic pain through the release of pro-inflammatory molecules [84], as shown in Figure 4. Emerging biomarkers for early detection and monitoring of DPN shown in Table 3.

### 6.3. Advances in Proteomics and Transcriptomics in Identifying New Targets

The proteomics and transcriptomics combination is revolutionizing our understanding of diabetic neuropathy. CQYG therapy by reducing protein processing in the endoplasmic reticulum caused decreased apoptosis, inflammatory factor expression, and mitochondrial damage [85]. Proteomic analysis revealed that the peripheral nerves of db/db mice had different expressions of proteins that were grouped into functional clusters, in comparison to control mice. These findings showed decreased metabolism of the glycolytic and TCA cycles, increased lipid catabolism, activation of the inflammatory response and acute phase, along with alterations in glutathione metabolism and proteins associated with oxidative stress. Inhibitors of molecular chaperones such as heat shock proteins 90 (HSP90) by KU-32 also showed an enhancement in both morphological and physiological indicators of degenerative neuropathy in some studies [86]. MicroRNA-146a (miR-146a) mimics and decreases TNF-receptor-associated factor 6 (TRAF6) and IL-1 receptor-associated kinase 1 (IRAK1) can be used as a new therapeutic target [87]. Inversely, downregulating miR-146a showed TNF-α, IL-1β production, and a loss of NF-κB inhibition in the sciatic nerve of DPN rats [87].

## 7. Conclusions

Diabetic peripheral neuropathy (DPN) is a severe consequence of diabetes that is caused by cytokine-driven processes and persistent inflammation that damage peripheral nerves. A vicious cycle of neuroinflammation and nerve degeneration is produced by pro-inflammatory cytokines such as TNF-α, IL-1β, and IL-6 that increase oxidative stress, interfere with Schwann cell function, and damage axonal integrity. Advances in understanding these pathways have highlighted cytokine signaling as a critical therapeutic target. Emerging interventions, from monoclonal antibodies to biomarker-driven strategies, offer new hope for mitigating inflammation and preserving nerve function. Future treatments that target the inflammatory causes of DPN may improve patient outcomes, highlighting the significance of ongoing studies into cytokine regulation as a fundamental component of novel therapeutic strategies.

## Figures and Tables

**Table 1:** Pro-inflammatory cytokines in the pathogenesis of diabetic peripheral neuropathy.

Cytokine	Source	Key Pathogenic Roles	Impacted Pathways
TNF-α	Macrophages, T-cells	Induces oxidative stress, Schwann cell dysfunction, and neuronal injury	NF-κB, MAPK
IL-1β	Monocytes, Neutrophils	Amplifies inflammatory response, promotes neuronal hyperexcitability	NF-κB, JAK/STAT
IL-6	Monocytes, Fibroblasts	Sustains pro-inflammatory environment, increases pain sensitivity	JAK2/STAT3, ERK
IL-17	T-helper 17 cells	Drives chronic inflammation, induces Schwann cell apoptosis	MAPK, NF-κB
IL-18	Monocytes, Macrophages	Enhances IFN-γ production, promotes chemokine release	NF-κB, MAPK

**Table 2:** Current therapeutic strategies for targeting cytokine signaling in DPN.

Therapy	Mechanism of Action	Preclinical/Clinical Evidence
Monoclonal antibodies (e.g., anti-TNF)	Inhibit TNF-α activity, reducing inflammation and nerve damage	Reduced pain and improved nerve function in preclinical models
Resveratrol	Activates Nrf2, suppresses oxidative stress and pro-inflammatory cytokine production	Improved sensory nerve conduction and reduced pain in animal studies
Melatonin	Reduces neuroinflammation via NF-κB suppression and oxidative stress modulation	Decreased COX-2 and TNF-α levels in diabetic rodent models
Progesterone	Reduces inflammation and edema around peripheral nerves	Neuroprotective effects demonstrated in animal models
TLR9 inhibitors	Suppress Toll-like receptor activity, reducing neuroinflammatory signaling	Significant reduction in neuropathic pain in rodent studies

**Table 3:** Emerging biomarkers for early detection and monitoring of DPN.

Biomarker	Type	Diagnostic/Therapeutic Relevance
TNF-α	Pro-inflammatory cytokine	Correlates with nerve conduction deficits and neuropathic symptoms
Advanced Glycation End-Products (AGEs)	Metabolic by-product	Linked to oxidative stress and neuronal injury; early diagnostic marker
CXCL10	Chemokine	Elevated levels associated with neuropathic severity
Nitrotyrosine (NT)	Oxidative stress marker	Indicative of nerve damage severity in diabetic patients
Adiponectin	Adipokine	Higher levels observed in DPN, suggesting potential as a biomarker

## Abbreviations

The following abbreviations are used in this manuscript:

COX-2	Cyclooxygenase-2
CRP	C-reactive protein
CSF-1	Colony Stimulating Factor-1
DPN	Diabetic peripheral neuropathy
FADD	Fas-associated death domain
G-CSF	Granulocyte-colony stimulating factor
IKK	IκB kinase
iNOS	Inducible nitric oxide synthase
JNK	c-Jun NH2-terminal kinases
MAPK	Mitogen-activated protein kinase
MAPKAPK2	Protein kinase-activated protein kinase 2
NGF	Nerve Growth Factor
NF-κB	Nuclear factor-kappa B
pDPN	Painful diabetic peripheral neuropathy
PRRs	Pattern recognition receptors
sICAM	Soluble intercellular adhesion molecule
sVCAM	Soluble vascular cell adhesion molecule
TLRs	Toll-like receptors
TNF-α	Tumor necrosis factor-alpha
TGF-β	Transforming growth factor β
TRADD	TNF receptor-associated death domain
TRAF-2	TNF-R-associated factor 2
